# *Candida albicans* as Marker of the Impact of Antibiotics on Gut Microbiome

**DOI:** 10.3390/pathogens15080832

**Published:** 2026-08-08

**Authors:** Afroditi Ziogou, Petros Ioannou, Andreas G. Tsantes, Georgia Vrioni, George Samonis

**Affiliations:** 1 Department of Internal Medicine, 417 Army Equity Fund Hospital, 115 21 Athens, Greece; 2 Department of Internal Medicine, University Hospital of Heraklion, 710 03 Heraklion, Greece; 3 Laboratory of Haematology and Blood Bank Unit, Attikon University Hospital, School of Medicine, National and Kapodistrian University of Athens, 124 61 Athens, Greece; andreas.tsantes@yahoo.com; 4 Department of Microbiology, “Saint Savvas” Oncology Hospital, 11522 Athens, Greece; 5 Department of Microbiology, School of Medicine, National and Kapodistrian, University of Athens, 115 25 Athens, Greece; gvrioni@med.uoa.gr; 6 First Oncology Department, Metropolitan Hospital, Neon Faliron, 185 47 Athens, Greece

**Keywords:** *Candida albicans*, gut microbioma, intestinal microbioma, antibiotics, antimicrobial agents, gastrointestinal tract

## Abstract

Background: *Candida albicans* constitutes part of the normal human gastrointestinal (GI) microbiome(GM). Alterations inthe GM induced by antibiotics may disrupt colonization resistance and promote fungal overgrowth. This review summarizes the effects of antibiotics on GI *C. albicans* colonization in experimental animal models and humans. Methods: A narrative review was conducted using data from the PubMed/MEDLINE and Scopus databases. Studies evaluating changes in GI *C. albicans* populations following antibiotic administration in mice or humans were included. Results: Twenty-six articles met the inclusion criteria, comprising 16 murine and 10 human studies. Across all studies, antibiotics were consistently associated with increased GI *C. albicans* colonization. The greatest increases were observed with broad-spectrum agents with activity against anaerobic bacteria. Elevated fungal concentrations frequently persisted after treatment discontinuation. Human studies largely reproduced findings from murine models. Despite substantial increases in GI colonization, dissemination beyond the GI tract was uncommon. Conclusions: Available evidence demonstrates that antibiotic-induced disruption of the GM promotes GI overgrowth of *C. albicans*. Hence, *C. albicans* concentration can serve as an indicator of antibiotics’ impact on the GM. While increased colonization alone rarely results in invasive disease, antibiotic-associated yeast expansion may represent an important step in the pathogenesis of disseminated candidiasis.

## 1. Introduction

The human gastrointestinal (GI) tract consists of a complex ecosystem of microorganisms collectively known as the gastrointestinal microbiome (GM), functioning as a metabolically active organ that contributes to host physiology and health. Its role is crucial in nutrient metabolism, vitamin synthesis, maintenance of intestinal barrier integrity, regulation of immune responses, and protection against colonization by pathogenic microorganisms [1]. Disruptions in GM composition and function, including reduced microbial diversity or altered abundance of key microbial taxa, a condition commonly referred to as dysbiosis, have been associated with numerous GI, metabolic, inflammatory and infectious diseases [2]. Although bacteria constitute the predominant component of the GM, the fungal community has emerged as an essential contributor to intestinal homeostasis and balance, referred to as the gut mycobiome. Fungi account for a relatively small proportion of the total microbial biomass; however, they are engaged in extensive interactions with bacterial communities and the host immune system, thereby influencing both local and systemic immune responses [3]. Among the fungal species detected in the GI tract, *C. albicans* is the most prevalent and extensively studied. This yeast is often documented as a commensal microorganism in healthy individuals and constitutes a normal component of the human GM [4]. As a member of the normal GM, *C. albicans* is typically maintained at low abundance through competitive interactions with commensal bacterial populations, particularly obligate anaerobic bacteria that contribute to intestinal colonization resistance. Under normal conditions, its growth depends on complex interactions with commensal bacteria, the intestinal mucus layer, epithelial barrier function, antimicrobial peptides and host immune surveillance [5]. 

The concept of colonization resistance describes the ability of the GM to prevent excessive proliferation of opportunistic microorganisms, including *C. albicans* [4]. Disruption of these bacterial communities, especially after exposure to broad-spectrum antibiotics that reduce the diversity of protective anaerobic commensals, weakens colonization resistance and creates favorable conditions for fungal expansion. As a result, increased intestinal colonization by *C. albicans* is frequently observed and may serve as a biological indicator of GM disruption and intestinal dysbiosis [6]. Likewise, corticosteroids may impair innate and adaptive immune responses, reducing the host’s ability to control fungal growth [7]. Cytostatic agents, whichpractically act as antibiotics, can also lead to microbial dysbiosis through mucosal injury, alterations in microbial composition,and suppression of immune defences [7]. Given that *C. albicans* is highly responsive to changes in the microbial GM balance and host immune status, this yeast has been proposed as a useful biological indicator of intestinal dysbiosis. Increased intestinal colonization by *C. albicans* has been consistently observed following exposure to antibiotics, corticosteroids and cytostatic drugs, reflecting disturbances in the normal equilibrium between bacterial and fungal communities [3,4]. 

In the present review, *C. albicans* was selected as an indicator of GM imbalance exposing the impact of specific antibiotics, corticosteroids and cytotoxic agents on gut microbial homeostasis and the mechanisms that promote fungal overgrowth. Understanding these interactions may provide valuable insights into the pathogenesis and clinical consequences of drug-induced intestinal dysbiosis. The present study is a narrative review of the available evidence regarding the effects of antibiotics on intestinal *C. albicans* colonization and the associated alterations in gut microbial homeostasis. Direct investigation of gut fungal population in humans is limited; thus, particular emphasis was placed on experimental studies employing murine models of GI *C. albicans* colonization. Additional evidence regarding the effects of corticosteroids and cytostatic agents on intestinal fungal populations was also reviewed where available.

## 2. Materials and Methods

### 2.1. Search Strategy and Inclusion and Exclusion Criteria

A targeted literature search was independently conducted by two authors (A.Z., G.S.) using the PubMed/MEDLINE and Scopus databases. Any disagreements regarding study eligibility were resolved through discussion and consensus among the investigators. The search included relevant records published until 5 June 2026. Data extraction was performed according to a predefined protocol. Search terms included combinations of “*Candida albicans*”, “gastrointestinal tract”, “gut microbiota”, “gut microbiome”, “intestinal colonization”, “fungal colonization”, “yeast colonization”, “gastrointestinal colonization”, “antibiotics”, “antimicrobial agents”, “antimicrobial therapy”, “corticosteroids”, “cytotoxic agents” and “mouse model”, “animal model”, “murine model” or “humans”. Eligible studies included original research articles reporting the effects of antibiotics, corticosteroids or cytostatic agents on GI *C. albicans* colonization. Both animal and human studies were considered. Review articles, editorials, conference abstracts, animal studies unrelated to GI colonization and publications lacking an accessible full text were excluded. Only articles published in English were considered eligible for inclusion. Additionally, the reference lists of included articles were manually screened to identify further relevant studies that may not have been captured through the electronic database search.

### 2.2. Murine Models Used for the Evaluation of Candida albicans Gut Colonization

For the evaluation of the effects of antibiotics on intestinal *C. albicans* populations, the present review included studies performed in murine models of *Candida* GI colonization. In most studies, the model used involved healthy adult male mice with normal GM fed supplemented *C. albicans* chow for 14 days, resulting in stable, constant and sustained colonization of the murine GI tract by the yeast, as documented by quantitative stool cultures. Following the feeding period, in this murine model, the yeast remained detectable in the GI tract at concentrations ranging from 3 to 4 log10 colony-forming units (CFUs)/g of stool and persisted at these concentrations for up to five months. Moreover, selected mice underwent microbiological culture and histopathological examination of various organs at the end of the study period; no evidence of tissue invasion or systemic organ infection by *Candida* species was detected [8]. Since, in this model, the fungal burden in this murine model remains relatively stable under baseline conditions, increases in the absolute fecal abundance of *C. albicans*, measured as CFU/g of stool by quantitative cultures, documented by quantitative cultures following drug administration can be interpreted as evidence of disrupted colonization resistance and GM imbalance. Several observations initially obtained with this murine model were subsequently investigated by the same researchers in human subjects, in whom fecal *Candida* concentrations were measured before and after antimicrobial treatment to assess the impact of antibiotics on the intestinal microbial ecosystem of the human gut. In another case, a different murine model has been used for the evaluation of the antibiotic effect on yeast GI colonization. In this experimental model, mice were first exposed to antibiotics to disrupt the indigenous GM and subsequently inoculated with *C. albicans*. This approach enabled the investigators to evaluate the effects of different antimicrobial agents on the establishment of GI colonization and the potential dissemination of the yeast from the GI tract under conditions of reduced bacterial colonization resistance [9,10]. A third similar experimental approach was employed by other researchers, in which mice were pre-treated with cefoperazone prior to oral inoculation with *C. albicans*, thus enabling the investigation of antibiotic-induced alterations in intestinal *Candida* colonization in relation to microbiota-derived metabolites [11]. Studies employing all three experimental approaches were included in the present narrative review that evaluates changes in the absolute GI abundance of *C. albicans* colonization following antibiotic administration and the associated alterations in gut microbial equilibrium. 

## 3. Results

### 3.1. Evidence from Murine Studies 

In total, 16 murine studies investigating the relationship between antibiotic exposure and GI colonization by *C. albicans* were identified [9,11,12,13,14,15,16,17,18,19,20,21,22,23,24,25]. All studies used established mouse models of intestinal stable and prolonged *C. albicans* colonization and examined the effects of antimicrobial agents on fungal burden within the GM [8,10,11]. The assessed outcomes included changes in stool *Candida* concentrations, persistence of *Candida* colonization following treatment discontinuation, dissemination to extra-intestinal organs, and development of systemic candidiasis.

A wide range of antimicrobial classes was investigated, including penicillin, cephalosporins, carbapenems, macrolides, tetracyclines, fluoroquinolones, glycopeptides, aminoglycosides, lipopeptides and oxazolidinones. Ceftriaxone was the most frequently studied antibiotic, appearing in four studies (25%). Carbapenems (ertapenem, imipenem, meropenem and doripenem), aminoglycosides, glycopeptides and macrolides were evaluated in multiple investigations, while the remaining agents were assessed in single studies. Across all studies, antibiotic administration resulted in increased GI colonization by *C. albicans*, although the magnitude of fungal expansion varied substantially between antimicrobial agents (Table 1). The highest fungal burdens were generally observed following administration of broad-spectrum agents with significant activity against anaerobic intestinal bacteria.

Among studies reporting quantitative stool counts, ticarcillin–clavulanic acid produced the highest fungal concentration, reaching 8.70 log10 CFU/g of stool at day 10 and remaining elevated at 7.1 log10 CFU/g one week after treatment cessation. Similarly pronounced increases were observed with tetracycline (8.46 log10 CFU/g), chloramphenicol (8.36 and 7.88 log10 CFU/g at day 10 and one week later, respectively), cefoperazone (8.2 and 5.0 log10 CFU/g) and amoxicillin–clavulanic acid (7.74 and 7.3 log10 CFU/g). Cefotaxime and ceftriaxone were also associated with marked fungal overgrowth, both reaching peak stool concentrations of approximately 7.2–7.3 log10 CFU/g and maintaining concentrations above 6 log10 CFU/g one week following treatment discontinuation. Metronidazole likewise resulted in substantial fungal expansion, with peak concentrations of 7.23 log10 CFU/g.

Among carbapenems, ertapenem produced the highest fungal burdens, reaching 6.7 log10 CFU/g at day 10 and 5.9 log10 CFU/g one week after treatment. Combination regimens including amikacin further enhanced fungal colonization, with ertapenem plus amikacin producing the highest fungal burden among carbapenem-containing regimens (7.12 log10 CFU/g at day 10). Imipenem and meropenem demonstrated slightly lower values, whereas doripenem exhibited the smallest effect among the carbapenems evaluated. Trimethoprim–sulfamethoxazole (TMP-SMX) was also associated with fungal expansion, resulting in stool concentrations of 6.81 log10 CFU/g at day 10 and 5.79 log10 CFU/g one week after treatment discontinuation.

Fluoroquinolones demonstrated comparatively modest effects. Ciprofloxacin produced the greatest increase within this class, followed by moxifloxacin, prulifloxacin and levofloxacin, which resulted in peak fungal burdens ranging from 5.23 to 5.52 log10 CFU/g and maintained similar concentrations one week after treatment discontinuation. Lower fungal burdens were observed with cefepime, cefixime and ceftibuten, which did not exceed 5.2 log10 CFU/g at day 10. Daptomycin had the lowest impact on fungal burden among quantitatively assessed agents, reaching 4.6 log10 CFU/g at day 10 and 4.2 log10 CFU/g one week later.

Taken together, several studies reported statistically significant increases in GI *C. albicans* colonization following antibiotic administration. Significant increases were observed with clindamycin, penicillin, vancomycin, tetracycline, ceftriaxone, cefoperazone, ticarcillin–clavulanic acid, chloramphenicol, ciprofloxacin, ertapenem, tigecycline and the fluoroquinolones levofloxacin, moxifloxacin and prulifloxacin. In addition, all tested macrolides, including erythromycin, clarithromycin, roxithromycin and azithromycin, were related to significantly increased stool fungal concentrations at the end of treatment.

Despite the marked increases in GI fungal burden, dissemination beyond the GI tract was uncommon. Fifteen out of the sixteen studies assessed fungal recovery from extra-intestinal sites, including the liver, spleen, kidneys, lungs and bloodstream. In 13 studies, no evidence of dissemination was detected despite substantial intestinal overgrowth. Only one study by Kennedy et al. reported dissemination, while another study examining ceftriaxone plus cyclophosphamide described colonization of extra-intestinal organs without histopathological evidence of tissue invasion [9,21]. Notably, in a study combining ceftriaxone administration with cyclophosphamide-induced immunosuppression, markedly elevated GI *C. albicans* concentrations were accompanied by fungal growth in extra-intestinal sites, including the lungs and spleen. These findings suggest that antibiotic-induced expansion of intestinal *C. albicans* is not sufficient by itself to induce invasive disease in immunocompetent hosts; dissemination appears to require additional predisposing factors such as immunosuppression [21]. A concise overview of all murine studies, including the antibiotics evaluated, stool *C. albicans* concentrations during and after treatment, statistical significance and dissemination to other organs, is presented in Table 2.

### 3.2. Evidence from Human Studies

A total of 10 clinical studies examined the effects of antibiotic administration on GI colonization by *C. albicans* in humans [26,27,28,29,30,31,32,33,34,35]. Collectively, these studies included 260 patients receiving a wide range of antimicrobial agents, including cephalosporins, penicillin, carbapenems, fluoroquinolones, tetracyclines, macrolides and combination regimens. Following the findings obtained from studies in mice, similar alterations in gut *Candida* colonization were observed in humans by measuring stool *C. albicans* concentrations before, during and after antimicrobial administration. In most studies, each patient served as his or her own control, with fungal colonization assessed prior to antibiotic exposure and compared with concentrations measured at day 10 of therapy and one week following antibiotic discontinuation. Overall, antibiotic administration was associated with increased GI colonization by *C. albicans* in most patient populations (Table 3). Similarly to the findings observed in murine models, the magnitude of fungal expansion varied considerably depending on the antimicrobial agent employed. Broad-spectrum β-lactams generally produced the greatest increases in fungal burden, whereas narrower-spectrum agents and antibiotics with limited effects on anaerobic intestinal microbiome led to more modest changes.

Unlike the murine studies, where ticarcillin–clavulanic produced the highest recorded stool fungal concentrations, the greatest increase observed in humans occurred following amoxicillin–clavulanate administration, which increased stool concentrations from 5.3 to 8.9 log10 CFU/g; elevated concentrations persisted one week after treatment discontinuation (6.7 log10 CFU/g). High fungal burdens were also observed following administration of levofloxacin, which resulted in peak concentrations of 8.2 log10 CFU/g and remained elevated at 7.7 log10 CFU/g one week after treatment cessation. Similarly, TMP-SMX and ofloxacin produced peak stool concentrations of 7.9 log10 CFU/g, while ciprofloxacin reached 7.3 log10 CFU/g during therapy.

Among β-lactam antibiotics, ceftriaxone was consistently associated with marked fungal expansion, increasing from 3.7 log10 CFU/g at baseline to 6.9 log10 CFU/g during treatment. Cefoperazone was likewise consistently associated with increased GI *Candida* colonization and, in comparative studies, generally produced greater fungal expansion than several other β-lactam agents, including cefoxitin, piperacillin and aztreonam. Amoxicillin and norfloxacin were associated with intermediate fungal burdens, reaching peak concentrations of 6.3 log10 CFU/g. Ticarcillin–clavulanate, moxifloxacin, ceftazidime and clarithromycin resulted in more modest increases, with peak concentrations ranging between 5.3 and 5.8 log10 CFU/g.

Carbapenems demonstrated moderate effects on intestinal colonization. Meropenem produced greater fungal expansion than cefepime in comparative studies, although both agents remained associated with persistent GI colonization during treatment. Imipenem–cilastatin and aztreonam resulted in peak concentrations of approximately 5.0 log10 CFU/g, whereas lower concentrations were observed with cefepime. Cephalosporins exhibited heterogeneous effects overall; while ceftriaxone consistently induced substantial fungal overgrowth, cefamandole, cefuroxime and cefoxitin produced comparatively small increases in stool *Candida* concentrations. Tetracycline-containing regimens were likewise associated with increased fungal colonization. Doxycycline alone produced moderate increases in stool *Candida* concentrations, whereas the combination of doxycycline and metronidazole resulted in the greatest fungal expansion within that study. Metronidazole administered as monotherapy exerted comparatively modest effects on fungal burden. Among all quantitatively assessed regimens, cefuroxime produced the lowest peak stool fungal concentration (2.8 log10 CFU/g). Of note, in the majority of human cases, although statistical analysis was not performed, the increase in *C. albicans* gut colonization could be considered statistically significant. 

Despite the consistent increase in GI *C. albicans* colonization observed across human studies included in this review, no cases of dissemination or invasive candidiasis were reported. All included investigations specifically noted the absence of systemic fungal infection, indicating that antibiotic-induced GI overgrowth alone is generally insufficient to cause invasive disease in otherwise immunocompetent individuals.

A concise overview of all human studies, including the antibiotics evaluated, stool *C. albicans* concentrations before and after antimicrobial treatment and dissemination findings, is presented in Table 4.

## 4. Discussion

This narrative review demonstrates a solid association between antibiotic exposure and increased GI colonization by *C. albicans* in both experimental mice models and human studies. Despite several differences in study design, patient populations, antimicrobial regimens and methods to quantify fungal burden, the overall findings were generally similar. In both mice and humans, administration of antibiotics was followed by an increase in GI *C. albicans* populations, with the greatest effects generally observed following exposure to broad-spectrum agents with substantial activity against anaerobic intestinal bacteria. Furthermore, elevated fungal burdens often persisted beyond treatment discontinuation, suggesting prolonged disruption of the GM. Notably, while antibiotic-induced fungal overgrowth was consistently observed, progression to disseminated candidiasis remained uncommon and was usually associated with additional host-related risk factors, including immunosuppression and impairment of mucosal barrier integrity. These findings support that antibiotic-induced dysbiosis plays a pivotal role in facilitating *C. albicans* expansion within the GI tract and may represent an important early step in the pathogenesis of invasive candidiasis.

The human GM constitutes a highly complex and dynamic microbial ecosystem that contributes to maintaining host homeostasis, influencing nutrient metabolism, immune system development and protection against pathogenic microorganisms. Colonization resistance constitutes one of its most important functions**,** impeding the establishment and expansion of opportunistic pathogens through competition for nutrients and ecological niches, production of antimicrobial metabolites, preservation of epithelial barrier integrity and modulation of both innate and adaptive immune responses [36,37,38]. Consequently, disruption of this balanced microbial community may significantly affect host susceptibility to intestinal colonization by opportunistic organisms, including *C. albicans* [38]. Recent advances in culture-independent sequencing technologies have demonstrated that reduced microbial diversity and depletion of obligate anaerobic bacteria are hallmarks of intestinal dysbiosis and are associated with impaired colonization resistance and increased pathogen expansion [39,40]. The findings of the present review are in accordance with these observations, demonstrating that antibiotic-induced perturbation of the GM represents a major determinant of GI *C. albicans* overgrowth. Notably, the greatest increases in fungal burden observed in both murine models and human studies followed administration of broad-spectrum antibiotics with activity against anaerobic intestinal bacteria, further explaining the pivotal role of the indigenous anaerobic GM in restricting *Candida* expansion within the GI tract.

Disruption of the GM extends beyond facilitating *Candida* overgrowth and has increasingly been recognized to affect a wide spectrum of adverse clinical outcomes. Loss of microbial diversity and depletion of beneficial commensal bacteria has been associated with impaired intestinal barrier function, deregulated immune responses and increased susceptibility to GI, as well as systemic infections [1]. Additionally, evidence indicates that the composition of the GM significantly influences the efficacy and toxicity of several therapeutic interventions. Regarding cancer patients, antibiotic-induced dysbiosis has been associated with reduced response to immune checkpoint inhibitors and less favorable outcomes in patients with solid tumors, highlighting the critical role of commensal microorganisms in modulating antitumor immunity [41,42]. Similarly, GM disruption has been implicated in hematopoietic stem cell transplantation, where reduced bacterial diversity is associated with an increased risk of bloodstream infections, graft-versus-host disease and elevated mortality rates [43,44]. Moreover, alterations in the GM have also been linked to *Clostridioides difficile* infection, inflammatory bowel disease, metabolic disorders and several immune-mediated diseases, emphasizing that preservation of microbial homeostasis is generally regarded as an important therapeutic goal [2,45]. As a result, antibiotic-induced yeast overgrowth should be considered as a manifestation of a broader disturbance of the intestinal ecosystem rather than an isolated phenomenon. 

The findings of the present review further indicate that the effect of antibiotics on GI *C. albicans* colonization is largely determined by their impact on the GM rather than by the antimicrobial spectrum alone. Broad-spectrum antibiotics with potent activity against obligate anaerobic bacteria consistently produced the greatest increase in stool *Candida* concentrations in both murine models and human studies. This observation particularly regarded β-lactam/β-lactamase inhibitor combinations, broad-spectrum cephalosporins, carbapenems, tetracycline and chloramphenicol, all of which resulted in marked fungal expansion in experimental models and, where available, in human studies [11,16,18,25]. In contrast, antibiotics with comparatively limited activity against the anaerobic bacteria generally produced smaller alterations in fungal burden. For instance, later-generation oral cephalosporins such as cefixime and ceftibuten, as well as daptomycin, exhibited only modest increases in GI *Candida* populations in murine models [11,18]. Similarly, although fluoroquinolones increased *C. albicans* colonization, their overall effect was generally lower than that observed with antibiotics exhibiting extensive anti-anaerobic activity; this finding may suggest that preservation of the indigenous anaerobic GM remains an important determinant of colonization resistance. These observations are consistent with experimental and clinical studies showing that obligate anaerobic bacteria belonging predominantly to the phyla *Bacillota* and *Bacteroidota* constitute the principal mediators of colonization resistance through the production of short-chain fatty acids, conversion of primary to secondary bile acids, competition for nutrients and modulation of mucosal immune responses [46,47,48].

Notably, our synopsis also suggests that not all broad-spectrum antibiotics exert equivalent effects on the GM. Among the broad-spectrum agents evaluated, TMP-SMX consistently produced comparatively modest increases in GI *C. albicans* colonization in both murine and human studies, despite its broad antibacterial spectrum [29]. This observation is biologically plausible, as TMP-SMX exhibits relatively limited activity against many obligate anaerobic bacteria that comprise the dominant GM, thereby preserving the microbial communities responsible for colonization resistance. Contrarily, antibiotics with activity against anaerobic organisms, including ticarcillin–clavulanate, amoxicillin–clavulanate, cefoperazone, ceftriaxone, carbapenems and tetracycline, were consistently associated with a greater fungal expansion in both animal and clinical studies. In total, these findings support that antimicrobial-induced disruption of anaerobic commensal bacteria, rather than antibacterial spectrum alone, is a key contributor to intestinal *C. albicans* overgrowth. However, the relationship between gut microbiota disruption and fungal colonization is likely bidirectional, with increasing *C. albicans* colonization also contributing to secondary alterations in GM composition. Consequently, when clinically appropriate, antibiotic administration strategies employing agents with a lower impact on the GM may contribute to colonization resistance and reduction inantibiotic-associated fungal overgrowth.

An additional objective of the present narrative review was to determine whether the antibiotic-induced increase in GI *C. albicans* colonization was followed by dissemination beyond the intestinal tract. This question is of particular clinical relevance since the GI tract has long been regarded as a potential source of invasive candidiasis. A landmark study by Krause et al. first demonstrated that orally administered *C. albicans* could cross the intestinal mucosa and be recovered from the bloodstream and urine of a healthy volunteer, suggesting that translocation from the gut to the systemic circulation is possible [49]. Nevertheless, our findings indicate that although antibiotics consistently promoted intestinal *C. albicans* overgrowth, dissemination to distant organs or systemic infection was uncommon among the studies included in this review. In both murine and human studies, despite increased intestinal fungal burden following antibiotic exposure, fungal dissemination was observed only rarely and generally required additional predisposing factors. Therefore, these findings should not be interpreted as indicating that antibiotic-associated candidiasis is rare in general, but rather that, in the available studies, intestinal *C. albicans*overgrowth alone rarely resulted in systemic dissemination; development of candidemia depends on the combined effects of increased intestinal fungal burden, disruption of mucosal barrier function and impaired host immune defenses [39,50,51]. 

The present review has several limitations that should be acknowledged. First, as a narrative review, it was not designed to systematically identify and quantitatively synthesize all available evidence, and therefore some relevant studies may have been inadvertently missed despite a comprehensive literature search. Secondly, the available evidence is based on a relatively limited number of experimental animal studies and clinical investigations, many of which included small sample sizes and differed considerably in study design, animal models, patient populations, antimicrobial regimens, treatment duration and methods used to quantify GI *C. albicans* colonization. Furthermore, this review integrates findings from heterogeneous preclinical and clinical study designs, which should be considered when interpreting overall conclusions. This methodological heterogeneity limited the possibility of drawing definitive conclusions regarding the relative impact of individual antibiotics. In addition, several studies lacked detailed reporting of baseline microbiological characteristics, statistical analyses or long-term follow-up, thereby restricting data interpretation. Furthermore, most studies evaluated changes in stool *C. albicans* concentrations as an indicator of intestinal colonization rather than directly characterizing the GM using contemporary sequencing techniques. Indeed, fungal colonization was predominantly quantified using culture-based methods, such as log10 CFU/g of stool, rather than contemporary microbiome profiling approaches like metagenomic sequencing. Moreover, stool-based measurements provide limited spatial resolution, rendering it difficult to determine whether changes in *C. albicans* colonization occur predominantly in the small or large intestine and may not accurately reflect mucosal colonization or the sites from which translocation and subsequent systemic dissemination originate. Consequently, alterations in bacterial community composition could only be inferred from fungal overgrowth and were not uniformly documented. Finally, only articles published in the English language were included, which may have introduced a degree of selection bias, although this is unlikely to have substantially influenced the overall conclusions of thisreview.

## 5. Conclusions

Antibiotic administration consistently promotes GI overgrowth of *C. albicans*, reflecting disruption of the GM and impairment of colonization resistance. Across both murine and human studies, the magnitude of fungal expansion varied substantially among antimicrobial agents and was greater following administration of antibiotics with activity against obligate anaerobic bacteria. In contrast, agents with a lower impact on the indigenous GM generally produced more limited alterations in *Candida* colonization. Importantly, although antibiotic-induced fungal overgrowth rarely progressed to disseminated infection in otherwise immunocompetent hosts, it represents an important indicator of GM disruption and may contribute to the development of invasive candidiasis in the presence of additional host-related risk factors. When clinically appropriate, narrow-spectrum antibiotics or agents associated with lower impact on the GM should be preferred over broad-spectrum regimens with extensive anti-anaerobic activity, thus minimizing disruption of colonization resistance while also maintaining adequate antimicrobial efficacy. Such an approach is particularly relevant in vulnerable patient populations, including individuals with malignancies, in whom intestinal dysbiosis has been associated not only with an increased risk of opportunistic infections but also with reduced responses to anticancer therapies.

Finally, increasing recognition of the pivotal role of the GM has led to the development of microbiome-preserving and microbiome-restoring therapeutic strategies. Approaches such as fecal microbiota transplantation and targeted microbial restoration may represent promising interventions for re-establishing colonization resistance following profound antibiotic-induced dysbiosis. Future studies integrating contemporary microbiome sequencing techniques with quantitative assessment of *Candida* colonization are needed to define the effects of individual antibiotics and to develop strategies that treat bacterial infections while preserving the integrity of the intestinal microbiome.

## Figures and Tables

**Table 1:** Antibiotics associated with the highest increase in stool *Candida* burden based on the maximum stool concentrations reported. Baseline *C. albicans* concentrations before treatment were 10^3^–10^4^ CFU/g of stool.

Antibiotic	Peak Stool *C. albicans* Concentration (log10 CFU/g Stool)
Ticarcillin–clavulanic acid	8.70
Tetracycline	8.46
Chloramphenicol	8.36
Cefoperazone	8.20
Amoxicillin–clavulanic acid	7.74
Cefotaxime	7.30
Ceftriaxone	7.20–7.30
Metronidazole	7.23
Ertapenem + amikacin	7.12
Imipenem + amikacin	6.82
Trimethoprim–sulfamethoxazole	6.81
Ertapenem	6.70
Ciprofloxacin	6.66
Imipenem–cilastatin	6.40
Tigecycline	6.30
Meropenem	6.20
Doripenem	5.90
Moxifloxacin	5.52
Prulifloxacin	5.23
Levofloxacin	5.23
Cefepime	5.20
Cefixime	5.00
Ceftibuten	4.90
Daptomycin	4.60

**Table 2:** Murine studies evaluating the effects of antibiotics on gastrointestinal *C. albicans*colonization.

Author	Year	Antibiotics Tested	Baseline Stool *C. albicans* Concentration Before Treatment (log10 CFU/g of Stool)	Peak Stool *C. albicans* Concentration Day 10 of Treatment/1 Week After Treatment (log10 CFU/g of Stool)	Difference in *C. albicans* Stool Concentration on Day 10 of Treatment/1 Week After Treatment (log10 CFU/g of Stool) (log10 CFU/g of Stool)	Statistically Significant Increase	Dissemination
Kennedy et al. [10]	1985	Penicillin, clindamycin, vancomycin, erythromycin, gentamicin	NR	NR	NR	Clindamycin, penicillin, vancomycin	Yes (highest with clindamycin)
Samonis et al. [24]	1990	Ceftriaxone	4.2	7.3/6.4	3.1/2.2	NR	None
		Cefoperazone		8.2/5	4/0.8		
		Ceftazidime		6.9/4.8	2.7/0.6		
		Ticarcillin/clavulanic acid		8.7/7.1	4.5/2.9		
		Imipenem-cilastatin		6.3/4.9	2.1/0.7		
		Gentamicin		6.2/4.6	2/0.4		
		Aztreonam		5.3/3.8	1.1/−0.4		
Samonis et al. [23]	1994	Cefotaxime	4.3	7.3/6.9	3/2.6	NR	None
		Amikacin		6.3/4.8	2/0.5		
		Pefloxacin		6.5/5	2.2/0.7		
		Amoxillin		5.9/4.9	1.6/0.6		
Samonis et al. [17]	1994	Amoxicillin-clavulanic acid	4.4	7.7/7.3	3.3/2.9	Chloramphenicol, Ciprofloxacin	None
		Chloramphenicol		8.4/7.9	4/3.5		
		Ciprofloxacin		6.7/5.5	2.3/1.1		
		TMP-SMX		6.8/5.8	2.4/1.4		
		Ampicillin		6/5.98	1.6/1.58		
Samonis et al. [22]	1994	Tetracycline	4.2	8.5/7.8	4.3/3.6	Tetracycline	None
		Metronidazole		7.2/6.3	3/2.1		
		Norfloxacin		6.5/5.8	2.3/1.6		
Samonis et al. [21]	1996	Ceftriaxone	4.4	7.3/NR	NR	Ceftriaxone, Ceftriaxone + Cyclophosphamide	Lung, spleen colonization in 2 mice, without tissue invasion
		Ceftriaxone+ Cyclophosphamide		7.3/NR			
Maraki et al. [19]	1998	Cefipime	4.2	5.2/4.6	1/0.4	Ceftriaxone	None
		Cefixime		5/4.7	0.8/0.5		
		Ceftibuten		4.9/4.4	0.7/0.2		
		Ceftriaxone		7.2/6.3	3/2.1		
Maraki et al. [25]	1998	Methylprednisolone	4.2	4.4/4.0	0.2/−0.2	Ceftriaxone, Ticarcillin- clavulanic acid	None
		Ceftriaxone		7.1/6.4	2.9/2.2		
		Methylprednisolone + Ceftriaxone		7.3/6.9	3.1/2.7		
		Ticarcillin-Clavulanic acid		8.0/6.8	3.8/2.6		
		Ticarcillin-Clavulanic acid + Methylprednisolone		8.2/7.0	4/2.8		
Maraki et al. [20]	1999	Ceftriaxone	4.2	7.3/6.9	3.1/2.7	All agents	None
		Dexamethasone		6.5/5.6	2.3/1.4		
		Ceftriaxone+ Dexamethasone		7.5/7	3.3/2.8		
Samonis et al. [16]	2002	Erythromycin	4.2	6.2/5.5	2/1.3	All macrolides at day10 of treatment	None
		Clarithromycin		5.6/5	1.4/0.8		
		Roxithromycin		5.4/4.8	1.2/0.6		
		Azithromycin		5.7/5.2	1.5/1		
Samonis et al. [12]	2006	Ertapenem	4.2	6.7/5.9	2.5/1.7	Ertapenem	None
		Imipenem		6.2/5.2	2/1		
		Meropenem		6.1/5.4	1.9/1.2		
Samonis et al. [15]	2006	Vancomycin	4.2	5.4/4.7	1.2/0.5	None	None
		Teicoplanin		5.5/4.9	1.3/0.7		
		Linezolid		5.1/4.6	0.9/0.4		
		Quinupristin-dalfopristin		6/5.1	1.8/0.9		
		Telithromycin		5.8/4.9	1.6/0.7		
Samonis et al. [14]	2008	Tigecycline	4.2	6.3/5.2	2.1/1	Tigecycline	None
		Daptomycin		4.6/4.2	0.4/0		
Maraki et al. [18]	2011	Levofloxacin	4.2	5.5/5.6	1.3/1.4	All agents	None
		Moxifloxacin		5.5/5.5	1.3/1.3		
		Prulifloxacin		5.2/5.3	1/1.1		
Samonis et al. [13]	2013	Imipenem	4.2	6.3/5.4	2.1/1.2	NR	None
		Imipenem+ amikacin		6.8/5.9	2.6/1.7		
		Meropenem		6.1/5.4	1.9/1.2		
		Meropenem+ amikacin		6.7/5.9	2.5/1.7		
		Ertapenem		6.7/5.9	2.5/1.7		
		Ertapenem+ Amikacin		7.1/6.2	2.9/2		
		Doripenem		6/5.2	1.8/1		
		Doripenem+ Amikacin		6.4/5.7	2.2/1.5		
Guinan et al. [11]	2019	Cefoperazone	NR	NR	NR	Cefoperazone	NR

TMP-SMX: trimethoprim–sulfamethoxazole, NR: not reported.

**Table 3:** Antibiotics associated with the highest increase in stool *Candida* burden in the human gut based on the maximum stool concentrations reported.

Antibiotic	Baseline Stool *C. albicans* Concentration (log10 CFU/g)	Peak Stool *C. albicans* Concentration (log10 CFU/g Stool)
Amoxicillin–clavulanate	5.3	8.9
Levofloxacin	6.3	8.2
TMP-SMX	7.3	7.9
Ofloxacin	5.9	7.9
Ciprofloxacin	4.0	7.3
Ceftriaxone	3.7	6.9
Amoxicillin	5.0	6.3
Norfloxacin	5.3	6.3
Ticarcillin–clavulanate	3.2	5.8
Moxifloxacin	6.0	5.6
Ceftazidime	3.6	5.4
Clarithromycin	4.4	5.3
Imipenem–cilastatin	4.2	5.0
Aztreonam	4.6	5.0
Doxycycline + metronidazole	2.96	4.79
Meropenem	2.8	4.8
Doxycycline	3.34	4.38
Amoxicillin + clarithromycin	5.7	4.2
Metronidazole	3.22	3.85
Cefamandole	2.8	3.8
Cefepime	2.9	3.6
Cefoxitin	1.1	3.6
Cefuroxime	1.9	2.8

TMP-SMX: Trimethoprim–sulfamethoxazole.

**Table 4:** Human studies evaluating the effects of antibiotics on gastrointestinal *C. albicans* colonization.

Author Name	Year	Number of Patients	Antibiotics Tested	Peak Stool *C. albicans* Concentration Before Treatment (log10 CFU/g of Stool)*	Peak Stool *C. albicans* Concentration Day 10 of Treatment/1 Week After Treatment (log10 CFU/g of Stool) *	Results	Dissemination
Mulligan et al. [35]	1982	4	Cefoperazone	NR	NR	Increase in *Candida* in all patients	NR
Giuliano et al. [34]	1987	20	Cefoxitin, piperacillin, cefoperazone,aztreonam	NR	NR	Highest increase with cefoperazone, then piperacillin, cefoxitin, no increase with aztreonam	NR
Samonis et al. [27]	1993	46	Ceftriaxone	3.7	6.9/ 5.3	Highest increase with ceftriaxone	None
			Ceftazidime	3.6	5.4/ 3.8		
			Ticarcillin–clavulanic	3.2	5.8/ 4.8		
			Imipenem–cilastatin	4.2	5.0/ 4.5		
			Aztreonam	4.6	5.0/ 3.5		
Samonis et al. [29]	1994	40	Amoxicillin– clavulanate	5.3	8.9/ 6.7	Highest increase with amoxicillin–clavulanate	None
			Ciprofloxacin	5.5	6.9/ 5.9		
			TMP-SMX	7.3	7.9/ 7.7		
			Ampicillin	6.3	6.8/ 6.3		
Thomakos et al. [26]	1998	28	Cefamandole	2.8	3.8/ 3.7	Highest increase with cefoxitin, not statistically significant	None
			Cefuroxime	1.9	2.8 /2.7		
			Cefoxitin	1.1	3.6/ 3.0		
Samonis et al. [30]	2001	20	Cefipime	2.9	3.6/ 2.6	Higher increase with meropenem	None
			Meropenem	2.8	4.8/ 2.4		
Maraki et al. [31]	2001	33	Amoxicillin	5.0	6.3/ 5.6	Highest increase with amoxicillin—not statistically significant	None
			Clarithromycin	4.4	5.3/ 5.2		
			Amoxicillin+ Clarithromycin	5.7	4.2/ 3.9		
Mavromanolakis et al. [33]	2001	30	Norfloxacin	5.3	6.3/ 4.5	Highest increase with ciprofloxacin, not statistically significant	None
			Ciprofloxacin	4.0	7.3/ 3.8		
			Ofloxacin	5.9	7.9/ 5.4		
Maraki et al. [28]	2003	9	Doxycycline	3.34	4.38/ 3.71	Statistically significant increase with doxycycline+ metronidazole	None
			Metronidazole	3.22	3.85/ 3.34		
			Doxycycline+ Metronidazole	2.96	4.79/ 4.05		
Samonis et al. [32]	2005	30	Levofloxacin	6.3	8.2/7.7	Statistically significant increase with both agents	None
			Moxifloxacin	6.0	5.6/ 4.7		

TMP-SMX: trimethoprim–sulfamethoxazole, NR: not reported, *: Values are reported as maximum observed concentrations when ranges were available or as peak mean concentrations when studies reported mean ± SD only.

## Data Availability

No new data were created or analyzed in this study. Data sharing is not applicable to this article.

## Abbreviations

The following abbreviations are used in this manuscript:

GI	Gastrointestinal
GM	Gastrointestinal Microbiome
CFUs	Colony-Forming Units
TMP-SMX	Trimethoprim–Sulfamethoxazole
NR	Not Reported
